# Non-Invasive Brain Stimulation: An Interventional Tool for Enhancing Behavioral Training after Stroke

**DOI:** 10.3389/fnhum.2015.00265

**Published:** 2015-05-15

**Authors:** Maximilian J. Wessel, Máximo Zimerman, Friedhelm C. Hummel

**Affiliations:** ^1^Brain Imaging and Neurostimulation (BINS) Laboratory, Department of Neurology, University Medical Center Hamburg-Eppendorf, Hamburg, Germany; ^2^Institute of Cognitive Neurology (INECO), Buenos Aires, Argentina; ^3^Favaloro University, Buenos Aires, Argentina

**Keywords:** stroke, motor learning, non-invasive brain stimulation, motor recovery, TMS, tDCS, neurorehabilitation

## Abstract

Stroke is the leading cause of disability among adults. Motor deficit is the most common impairment after stroke. Especially, deficits in fine motor skills impair numerous activities of daily life. Re-acquisition of motor skills resulting in improved or more accurate motor performance is paramount to regain function, and is the basis of behavioral motor therapy after stroke. Within the past years, there has been a rapid technological and methodological development in neuroimaging leading to a significant progress in the understanding of the neural substrates that underlie motor skill acquisition and functional recovery in stroke patients. Based on this and the development of novel non-invasive brain stimulation (NIBS) techniques, new adjuvant interventional approaches that augment the response to behavioral training have been proposed. Transcranial direct current, transcranial magnetic, and paired associative (PAS) stimulation are NIBS techniques that can modulate cortical excitability, neuronal plasticity and interact with learning and memory in both healthy individuals and stroke patients. These techniques can enhance the effect of practice and facilitate the retention of tasks that mimic daily life activities. The purpose of the present review is to provide a comprehensive overview of neuroplastic phenomena in the motor system during learning of a motor skill, recovery after brain injury, and of interventional strategies to enhance the beneficial effects of customarily used neurorehabilitation after stroke.

## Background

Stroke is a leading cause of serious long-term disability (Kochanek et al., 2012) with growing impact on actual and future health economy. It is estimated that an additional four million people in the US will suffer a stroke by 2030, due to changes in demographic evolution (Heidenreich et al., 2011). This increase in the aging population will result in more demands on health services as stroke in older people often result in more severe functional loss (Baztan et al., 2007).

A large proportion of the focus of stroke research still remains on the acute management of stroke. The development of thrombolytic therapy, determination of an individualized time window to apply thrombolysis (Thomalla et al., 2011), and reduction in early post-stroke complications due to the application of the stroke unit concept, has led to a significant decline of mortality rate after stroke (Langhorne et al., 1993). On the other hand, a substantial proportion of stroke victims are left with moderate to severe disability. Indeed, the resulting motor deficit, especially of the upper extremity, has a great impact on activities of daily life. Currently, recovery of hand motor function in a large part of the survivors (55–75%) is unsatisfying (Nakayama et al., 1994; Jorgensen, 1996; Jorgensen et al., 1999).

As a result, there is a strong need for new innovative strategies to improve stroke rehabilitation. Comprehensive evidence indicates that motor learning mechanisms are operative during spontaneous stroke recovery and interact with rehabilitative training (Krakauer, 2006). As a matter of principle, re-acquisition of skills resulting in improved or more accurate motor performance is paramount for recovery of motor function after a brain lesion. Thus, to a significant degree, the success of neurorehabilitation depends on the amount and effectiveness of rehabilitative training to promote the re-acquisition of motor skill that were lost due to the lesion. In this context, non-invasive brain stimulation (NIBS) techniques have the appeal of being able to specifically and selectively enhance adaptive patterns of activity, suppress maladaptive patterns, and interact directly with the process of motor skill acquisition by sharing synergistic impacts on synaptic plasticity and network reorganization (Bolognini et al., 2009; Reis et al., 2009a; Fritsch et al., 2010).

Here, we review basic principles of motor learning. Further, we focus on modulation of its principles by NIBS and innovative training regimes.

## Principles of Motor Learning

**Figure 1:**
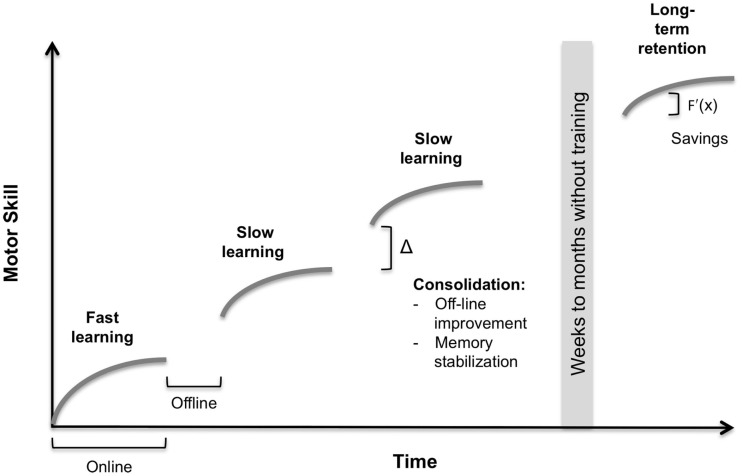
**Conceptual components in motor learning**. The illustrated learning curve indicates the increase in motor skill over time. Online – within-session learning. Offline – in-between-session learning. Fast learning – single-session practice. Slow learning – multiple-session practice. Consolidation – offline-improvement and memory stabilization. Long-term retention – skill retention after a prolonged interval. Savings – impact of previous learning on faster retraining, expressed in an increased slope *f*′(*x*) of the learning curve. For a detailed description, please see text.

Learning of a motor skill is commonly defined as a process of increased spatial and temporal accuracy of movements with practice (Willingham, 1998). The learning process consists of different temporal components; for illustration, please see Figure 1. In principle, online and offline processes can be segregated. Online learning refers to within-session, and offline learning to in-between session improvement (Robertson et al., 2004a).

During online learning, a first fast learning phase is characterized by rapid improvement usually within in a single training session. Whereas, in slow online learning further improvements develop over multiple sessions (Doyon and Benali, 2005). It is of note that the duration of both phases is highly dependent on task complexity (Dayan and Cohen, 2011). Procedural consolidation occurs after practice and incorporates two distinct processes offline-improvement and memory stabilization. Offline-improvement refers to in-between training session skill improvement. Memory stabilization results in diminished interference in memory encoding or retrieval by another consecutive task (Robertson et al., 2004a). Long-term retention describes the skill retention after a prolonged (e.g., 1 year) post-training time interval (Romano et al., 2010). An additional interesting concept is the term savings. First described in the motor adaptation literature, it is defined as the impact of previous learning on faster retraining in consecutive sessions (Landi et al., 2011).

The addressed learning components are represented by specific neuroanatomical substrates. Increased activity of the premotor cortex, supplementary motor area (SMA), parietal regions, striatum, and the cerebellum and decreased activity in the dorsolateral prefrontal cortex (DLPFC), primary motor cortex (M1), and presupplementary motor area (pre-SMA) have been associated with the fast learning progresses. Whereas, increased activation in primary motor cortex (M1), primary somatosensory cortex, SMA, and putamen and decreased activation in lobule VI of the cerebellum have been implicated with the slow learning process (Floyer-Lea and Matthews, 2005; Dayan and Cohen, 2011). Explicit learning has been shown to be sleep- and implicit learning time-dependent (Robertson et al., 2004b). The role of the M1 in offline learning has been disentangled with inhibitory NIBS. Inhibitory repetitive transcranial magnetic stimulation (rTMS) of M1 interfered with early consolidation (Muellbacher et al., 2002), but not with over-night improvements of a motor practice (Robertson et al., 2005). However, sleep-dependent motor consolidation has been associated with increased activation in the striatum (Debas et al., 2010).

These neurobehavioral concepts depend on neuroplastic changes within the brain. Motor learning is incorporated in molecular, cellular, and systemic substrates. An association with *de novo* protein synthesis has been shown (Luft et al., 2004). Several cellular mechanisms, for instance, modulation of synaptic efficacy (Rioult-Pedotti et al., 2000), change in neuronal membrane excitability (Halter et al., 1995), and anatomical changes like spine formation (Greenough et al., 1985) and axonal sprouting (Toni et al., 1999), among others have been described. Moreover, during motor skill learning, changes in its cortical representation have been demonstrated (Pascual-Leone et al., 1995).

Also on the systems level, neuronal networks undergo plastic changes. Influential integrative models have been proposed (Hikosaka et al., 2002; Doyon and Benali, 2005). Doyon and Ungerleider hypothesize in their model that in early learning, cortico-striatal and cortico-cerebellar networks are recruited. Interactions of these structures shape the acquired motor memory traces during the learning process (Ungerleider et al., 2002; Doyon et al., 2003).

## Neuroplastic Changes Observed after Stroke

Neuroplasticity after stroke and motor learning in the healthy brain share several common mechanisms. Described common neurobiological phenomena are axonal sprouting (Carmichael et al., 2001), dendritic remodeling (Brown et al., 2010), and reorganization of motor maps (Nudo and Milliken, 1996), among others. Furthermore, animal models could prove that rehabilitative training can influence the reorganization in the adjacent intact cortex (Nudo et al., 1996). For review, please see Hosp and Luft (2011) and Hallett (2001).

The neural correlates of stroke recovery have been described in several cross-sectional and longitudinal studies using functional neuroimaging (fMRI and PET) and TMS (Johansen-Berg et al., 2002; Ward et al., 2003; Gerloff et al., 2006; Lotze et al., 2006; Grefkes et al., 2008). Most of these studies revealed abnormal patterns of activation/excitability immediately after stroke and during the recovery process. Movement of the affected hand elicit a bilateral neural recruitment within motor areas in patients with subcortical lesions, which cannot be found in healthy subjects or when patients move the unaffected hand (Ward et al., 2003). In this regard, patients with good functional outcomes demonstrated a more lateralized neural activation pattern, while those patients whose motor deficit remained more severe recruited motor areas in both hemispheres (Ward et al., 2004). The significance of more widespread neural activation within the motor network during motor performance of the affected hand is still under debate. As recently discussed (Hummel et al., 2008a), the notion that the unaffected hemisphere has a non-beneficial effect on the lesioned hemisphere and consecutive behavior does not apply to all groups of patients. This fact might relevantly depend on lesion location, time after stroke, size, and integrity of the corticospinal pathway, among other factors (Lotze et al., 2006; Bradnam et al., 2011).

Patients, who suffered a stroke, exhibit changes in motor cortical excitability in both hemispheres (Shimizu et al., 2002; Cicinelli et al., 2003). Moreover, abnormal levels of inter-hemispheric inhibition from the unaffected to the affected motor cortex, resulting in an imbalanced inter-hemispheric interaction influence the function of the paretic hand (Murase et al., 2004) [for details regarding this concept, please see Schulz et al. (2013), Hummel and Cohen (2006), and Nowak et al. (2009)].

Following the concept of imbalanced inter-hemispheric inhibition, two main strategies for NIBS have been proposed: (1) NIBS is used to inhibit the motor cortex of the unaffected (contralesional – cM1) hemisphere to reduce the abnormal inhibitory drive toward the lesioned hemisphere. (2) An alternative approach is to facilitate the motor cortex of the affected (ipsilesional – iM1) hemisphere (Hummel and Cohen, 2006).

Additionally, neuroimaging studies have shown that premotor areas of the affected hemisphere are commonly more active during hand movement with the paretic hand after stroke, suggesting that non-primary motor areas of the same hemisphere are utilized when M1 is affected (Gerloff et al., 2006).

## Techniques for Non-Invasive Brain Stimulation

Non-invasive brain stimulation techniques can be used to influence cortical excitability, neuroplasticity, and behavior [for review, see Nitsche et al. (2008), Hummel and Cohen (2005)]. Excitatory or inhibitory plastic changes can be induced, depending on the used mode. Transcranial direct current stimulation (tDCS), transcranial magnetic stimulation (TMS), and paired associative stimulation (PAS) are the most common and widely used techniques (Hummel and Cohen, 2005).

By using tDCS small sub-threshold currents (1–2 mA) delivered via scalp electrodes are capable of influencing neuronal excitability by increasing or decreasing the respective transmembrane potentials. The induced changes are polarity specific. Anodal stimulation facilitates and cathodal stimulation inhibits motor cortex excitability (Nitsche et al., 2008). Advantages of tDCS are its simplicity and relative low cost. Limitations are its rather moderate temporal and focal resolution (Gandiga et al., 2006).

By adjusting the electrode placement (electrode montage), different cortical areas can be modulated. An active (target) electrode is placed over the target area and a reference (return) electrode over another cephalic or extracephalic region. The scalp position of electrode placement is usually defined by anatomical landmarks, the TMS hotspot, the 10/20 EEG system, or via stereotactical neuronavigation (Moos et al., 2012). It is important to note that the reference electrode is not physiologically inert (Nitsche et al., 2008). Recently, a framework for the categorization of tDCS montages has been proposed (Nasseri et al., 2015). Most tDCS studies in stroke rehabilitation so far used a bilateral bipolar non-balanced or balanced montage. In addition, first modeling studies, using finite element realistic head models, were able to calculate cortical current density distributions considering brain lesions (Wagner et al., 2007) and multifocal stimulation (Ruffini et al., 2014). This could help to find revised montages for future studies.

Transcranial magnetic stimulation uses the principle of electromagnetic induction. With a sufficient induced electrical field, it is possible to depolarize neurons. When the magnetic pulses are applied in a repetitive mode (rTMS), this results in excitability changes outlasting the duration of stimulation (Rossi et al., 2009). Conventional rTMS applied at a low frequency (0.2–1 Hz) results in inhibition. When it is applied at high frequency (≥5 Hz), it leads to excitation (Hallett, 2007). Recently, novel patterned rTMS protocols have been developed, the so-called theta-burst stimulation (TBS) (Huang et al., 2005). In the theta-burst protocol (TBS), three stimuli at 50 Hz are repeated at 5 Hz. In a continuous mode, this results in inhibition, in an intermittent mode in excitation (Hallett, 2007). Advantages of rTMS are its good spatial and temporal resolution, disadvantages are the relative complex and expensive setup and the rare but apparent occasion of relevant side effects, in particular seizures (Gandiga et al., 2006).

Paired associative stimulation is capable of inducing heterosynaptic plasticity. This is achieved by combining low-frequency peripheral nerve stimulation (PNS) with TMS to the motor cortex. The underlying concept is based on the idea that if both stimuli arrive at the same time (synchronous) at the cortex, an excitation will be induced. By contrast, asynchronous stimulation leads to inhibition (Stefan et al., 2000). Repetitive synchronous stimulation results in LTP-like effects, asynchronous stimulation in LTD-like effects. Advantages of PAS are that the protocol was directly developed on the basis of LTP/LTD-plasticity protocols of basic research and that many of its physiological properties are well studied. Disadvantages are its rather complex setup, its inter-individual variability, and that effects of protocol variation have not been systematically studied (Ziemann et al., 2008).

Recently, the repertoire of NIBS techniques has been enlarged by transcranial alternating current stimulation (tACS), transcranial random noise stimulation (tRNS), and more complex rTMS protocols (quadri- and octapulse rTMS). These methods have not been widely applied to enhance functional recovery after stroke, for detailed descriptions of these techniques, please see Antal and Paulus (2013), Terney et al. (2008), and Hamada and Ugawa (2010).

## Non-Invasive Brain Stimulation and Motor Learning in Healthy Subjects

First studies in healthy volunteers provided evidence that NIBS can cause transient behavioral effects in motor function (Wassermann et al., 1996; Boggio et al., 2006). Following this concept, first studies were designed to investigate whether NIBS can also modulate motor learning. The main target of most studies conducted so far was M1. In a first study by Nitsche et al. (2003), anodal tDCS applied to the contralateral M1 improved learning of an implicit task (Nitsche et al., 2003). By using an isometric-pinch task, Reis et al. (2009b) were able to demonstrate improved learning after five consecutive days of training with tDCS compared to sham, primarily driven by an offline (consolidation) effect. tDCS not only led to significant greater total learning but also the behavioral improvement remained superior in the tDCS group compared to sham even up to 3 months after training (Reis et al., 2009b). Anodal tDCS applied concurrently with practice demonstrated to enhance encoding and retention of a motor memory on a shorter time-scale. More recently, Tecchio et al. (2010) reported an improvement of early motor memory consolidation accomplished by anodal tDCS over the M1 in a serial finger-tapping task (Tecchio et al., 2010). Overall, there is accumulative evidence that tDCS is effective in promoting long-term plastic changes associated with learning and memory formation when increased excitability and changes in synaptic efficacy co-occur. It is of note that the facilitatory effect of anodal tDCS on learning might be task specific, depending on the state of cortical activation induced by the motor task (Bortoletto et al., 2014).

Furthermore, PAS modulated plasticity in healthy young subjects. PAS was paired with rapid thumb abduction movements, a basic model of motor learning; this led to the prevention of PAS_syn_-induced LTP-like plasticity. Moreover, when PAS_asyn_ was paired with this basic motor learning paradigm, subsequent PAS_asyn_-induced LTD-plasticity was enhanced (Ziemann et al., 2004). This provides evidence that PAS can induce use-dependent plasticity in healthy subjects and interacts with motor learning.

A revealing model to study altered motor learning networks is healthy aging. In a promising study, anodal tDCS applied to M1 concurrently to an explicit motor learning task resulted in substantial improvements during training (Zimerman et al., 2013). Furthermore, the anodal stimulation group showed superior performance at the 24-h follow-up compared to sham.

## Non-Invasive Brain Stimulation and Motor Learning in Stroke

The promising results of the discussed conceptual studies in young and old healthy subjects led to the interesting hypothesis of testing NIBS as an adjuvant therapy in neurological disorders. A resulting question was whether transient behavioral effects could be reproduced in stroke patients. The main conceptual target was to use NIBS for normalizing imbalanced inter-hemispheric inhibition. Following proof-of-principle studies demonstrated that anodal tDCS applied to iM1 and cathodal tDCS applied to cM1 improved transiently motor performance of the affected upper limb (Fregni et al., 2005; Hummel et al., 2005, 2006). Complementary rTMS-studies revealed that modulation of transcallosal inhibition with inhibitory 1 Hz rTMS to cM1 (Mansur et al., 2005; Takeuchi et al., 2005) and excitatory 20 Hz rTMS to iM1 improved motor function in chronic stroke patients (Yozbatiran et al., 2009).

As discussed in greater detail above, motor learning is essential for neurorehabilitation (Krakauer, 2006). In this regard, a pivotal question was whether NIBS could facilitate motor learning in patients. First studies investigated the effect of NIBS on motor learning in chronic stroke patients. For instance, cathodal tDCS applied to the cM1 during the performance of explicit motor learning task enhanced fast online acquisition yielding to a better task retention after 24 h. Interestingly, there was an association between tDCS-induced improvement during training and GABAergic intracortical changes in the affected motor cortex (Zimerman et al., 2012). Moreover, it could be shown that excitatory 10 Hz rTMS applied to iM1 immediately before each block of a sequential finger-tapping task, enhanced its acquisition in chronic stroke patients (Kim et al., 2006).

The main objective for translational research is to combine NIBS with repetitive occupational therapy, which uses principles of motor learning, in a clinical setting. For instance, Kim et al. paired 10 sessions of tDCS in a double-blind parallel design with occupational therapy of the upper limb in sub-acute stroke patients. Cathodal tDCS to cM1 resulted in superior motor function, assessed with the Fugl–Meyer Score, in a 6-month follow-up (Kim et al., 2010). In addition, excitatory rTMS to iM1 as add-on to normal physical therapy over 10 consecutive days in sub-acute stroke patients improved patients’ motor scores when compared to sham (Khedr et al., 2005). Bolognini and collaborators paired bihemispheric tDCS (cathodal stimulation to cM1 an anodal stimulation to iM1) with constraint-induced movement therapy (CIMT) over 10 treatment sessions in chronic stroke patients. Patients in the active group showed superior gains in hand function, measured with the Jebsen Taylor Hand Function Test (JTT), Handgrip Strength, Motor Activity Log Scale, and Fugl–Meyer Motor Score. Beyond that, as a neurophysiological correlate, the authors could identify a reduction in inter-hemispheric inhibition from the intact to the affected hemisphere, measured with double-pulse TMS (Bolognini et al., 2011). Another interesting concept, especially for patients with severe upper limb paresis, is the combination of tDCS with robot-assisted arm training. However, first promising results of a pilot study from Hesse et al. (2007) could not be replicated in a subsequent larger trial (Hesse et al., 2011). A possible explanation could be that the majority of the recruited patients had large cortical lesions and were severely affected. In a secondary analysis, patients with pure subcortical lesions improved significantly more after cathodal stimulation of the cM1 than patients with cortical involvement, pointing toward a need of individual patient stratification.

Further evidence that lesion location might have a crucial impact on the response to stimulation comes from a study conducted by Ameli et al. (2009). In their study, patients with subcortical stroke improved in dexterity after 10 Hz rTMS to iM1. This beneficial effect was not apparent in patients with additional cortical strokes (Ameli et al., 2009).

A compelling approach is dual-tDCS stimulation (anodal electrode over iM1 and cathodal electrode over cM1). Lefebvre et al. (2012) used this montage in chronic stroke patients with moderate deficits while training a complex visuomotor skill. A single-session of dual tDCS enhanced the online learning, leading to superior long-term retention (Lefebvre et al., 2012). Although promising, there is so far no clear scientific evidence that bifocal stimulation is more efficient than monofocal, or for which patients’ bifocal or monofocal might lead to more improvements. These are important questions, which have to be addressed in upcoming larger trials. Recently, novel training regimes, like virtual reality motor training, have been paired with tDCS in sub-acute stroke patients and have shown beneficial effects (Kim et al., 2014a).

In addition, first studies evaluated the feasibility of PAS in stroke patients. PAS_syn_ resulted in a significant facilitation of the extensor carpi radialis (ECR) MEP amplitude of the paretic side after 5 months following subcortical stroke. Partly, the facilitation was still present 12 months after the stroke. Furthermore, the neurophysiological changes were accompanied with improvements in the wrist section of the Fugl–Meyer motor scale and wrist force (Castel-Lacanal et al., 2007, 2009).

To date, a number of sham-controlled studies have been performed to investigate stimulation-associated enhancement of motor recovery after stroke (for detailed overview, please see Table 1). To evaluate the potential beneficial effects of NIBS, further multicenter clinical trials are needed. In this regard, the NETS-trial (Neuroregeneration Enhanced by Transcranial Direct Current Stimulation (tDCS) in Stroke, ClinTrialGov NCT009097 14) has been initiated. In the on-going study, anodal tDCS to iM1 combined with standard occupational therapy is applied in 10 consecutive sessions in sub-acute stroke patients.

**Table 1:** **Summary of the sham-controlled studies performed with non-invasive brain stimulation in motor recovery after stroke**.

	Number of patients	Cortical/subcortical	Ischemic/hemorrhagic	Severity of stroke	Stroke duration	Motor assessments and outcomes	Concomitant therapy	Study design	NIBS Intervention	Number of sessions	Follow-ups	Result
**rTMS**												
Khedr et al. (2005)	52	Cortico-subcortical	Ischemic	Moderate to severe	5–10 days	SSS, NIHSS, BI	Standard physical therapy	Randomized, parallel groups	3 Hz rTMS over iM1	10 days	10 days	Pos
Mansur et al. (2005)	Stroke 10, healthy 6	Cortico-subcortical	Ischemic	Mixed	<12 months	sRT, cRT, PP, FT	NA	Randomized, cross-over	1 Hz rTMS over cM1 and cPM	One	NA	Pos
Takeuchi et al. (2005)	20	Subcortical	Ischemic	Mixed	26.95 months	FM, PA	NA	Randomized, parallel groups	1 Hz rTMS over cM1	One	NA	Pos
Fregni et al. (2006)	15	Cortical 1, subcortical 13, cortico-subcortical 1	Ischemic	Mild to moderate	44.05 months	MRC, ASS, JTT, sRT, cRT, PPT	NA	Randomized, parallel groups	1 Hz rTMS over cM1	5 days	2 weeks	Pos
Kim et al. (2006)	15	Cortical 5, subcortical 10	Ischemic 12, hemorrhagic 3	Mild to moderate	16.7 months	PP, GF, FT	FT	Randomized, cross-over	10 Hz rTMS over iM1	One	NA	Pos
Liepert et al. (2007)	12	Subcortical (2 pons)	NM	Mild	7.3 days	MRC, GF, NHPT	NA	Randomized, cross-over	1 Hz rTMS over cM1	One	NA	Pos
Malcolm et al. (2007)	20	Cortico-subcortical	Hemorrhagic 1	Mixed	45.6 months	WMFT, BBT, MAL	CIT	Randomized, parallel groups	20 Hz rTMS over iM1	10 days	6 months	Neg
Talelli et al. (2007)	6	Cortical 3, subcortical 3	Ischemic	Mild to moderate	31 months	BI, NIHSS, ARAT, 9HP, GF, sRT, cRT	NA	Randomized, cross-over	iTBS over iM1, cTBS over cM1	One	NA	Pos
Dafotakis et al. (2008)	12	Subcortical	Ischemic	Mild	1.88 months	MRC (4–5), NIHSS, ARAT, grip-lift task	Grasping and lifting	Randomized, cross-over	1 Hz rTMS over cM1	One	NA	Pos
Mally and Dinya (2008)	64	Cortical, large	Ischemic, hemorrhagic 18	Severe	129.6 months	Spasticity score	NA	Randomized, parallel groups	1 Hz rTMS over cM1 and iM1	7 days	3 months	Pos
Nowak et al. (2008)	15	Subcortical	Ischemic	Mild	1.93 months	ARAT, MRC (4–5), FT, reach to grasp	NA	Randomized, cross-over	1 Hz rTMS over cM1	One	NA	Pos
Takeuchi et al. (2008)	20	Subcortical	Ischemic	Mixed	29.9 months	FM, acceleration and PF	PF training	Randomized, parallel groups	1 Hz rTMS over cM1	One	1 week	Pos
Ameli et al. (2009)	29	Cortical 13, subcortical 16	Ischemic	Mild to moderate	5.5 months	MRC, ARAT, mRS, NIHSS, index finger and hand tapping	NA	Randomized, cross-over	10 Hz rTMS over iM1	One	NA	Mix
Khedr et al. (2009)	36	Cortical 19, subcortical 17	Ischemic	Mild to moderate	0.57 months	MRC, NIHSS, BI, tapping, PP	NA	Randomized, parallel groups	1 Hz rTMS over cM1, 3 Hz over iM1	Five	3 months	Pos
Takeuchi et al. (2009)	30	Subcortical	Ischemic	Mixed	28.8 months	FM, acceleration and PF	Motor training (pinching task)	Randomized, parallel groups	1 Hz rTMS over cM1, 10 Hz over iM1, bilateral rTMS	One	1 week	Pos
Chang et al. (2010)	28	Cortical 11, subcortical 17	NM	Moderate to severe	13.4 days	MI, FM, GF, BB	Reaching and grasping exercises	Randomized, parallel groups	10 Hz over iM1	10	3 months	Pos
Emara et al. (2010)	60	Cortical 43, subcortical 17	Ischemic	Mild to moderate	>1 month	FT, mRS, AI	Standard physical therapy	Randomized, parallel groups	5 Hz rTMS over iM1, 1 Hz rTMS over cM1	Ten	12 weeks	Pos
Grefkes et al. (2010)	11	Subcortical	Ischemic	Mild	1.91 months	MRC (4–5), ARAT, NIHSS, whole hand fist task	NA	Randomized, cross-over	1 Hz rTMS over cM1	One	NA	Pos
Avenanti et al. (2012)	30	Cortical 3, cortico-subcortical 1, subcortical 26	Ischemic 20, hemorrhagic 10	Mild	31.47 months	JTT, NHPT, BB, PF	Standard physical therapy	Randomized, parallel groups	1 Hz rTMS over cM1	10	3 months	Pos
Chang et al. (2012)	17	Cortical 2, subcortical 15	Ischemic 14, hemorrhagic 3	Mild to moderate	>3 months	JTT, SFTT	SFTT	Randomized, parallel groups	10 Hz rTMS over iM1	10	1 month	Pos
Conforto et al. (2012)	30	Subcortical 16, cortical 14	Ischemic	Mild to severe	0.92 months	MRC, NIHSS, JTT, PF	Standard physical therapy	Randomized, parallel groups	1 Hz rTMS, over cM1	10	1 month	Pos
Seniow et al. (2012)	40	Cortical 16, subcortical 14	Ischemic 35, hemorrhagic 5	Moderate	<3 months	WMFT, NIHSS, FM	Standard physical therapy	Randomized, parallel groups	1 Hz over cM1	3 weeks	3 months	Neg
Wang et al. (2012)	28	NM	NM	Moderate	>6 months	FM, WP	Task-oriented training	Randomized, parallel groups	1 Hz rTMS over cM1	Ten	NA	Pos
Etoh et al. (2013)	18	Cortical 1, subcortical 17	Ischemic 13, hemorrhagic 5	Severe	29.9 months	FM, ARAT, MAS	Physical or occupational therapy	Randomized, cross-over	1 Hz rTMS over cM1	Ten	4 weeks	Pos
Higgins et al. (2013)	11	NM	NM	Mild to severe	>3 months	BB, WMFT, MAL, GF, PF, SIS	Task-Oriented Training	Randomized, parallel groups	1 Hz TMS over cM1	Eight	NA	Neg
Sasaki et al. (2013)	29	Subcortical	Ischemic 13, hemorrhagic 16	Mild to moderate	0.58 months	NIHSS, GF, FT	Standard physical therapy	Randomized, parallel groups	10 Hz rTMS over iM1, 1 Hz rTMS over cM1	Five	NA	Pos
Sung et al. (2013)	54	Cortical 35, subcortical 19	Ischemic 35, hemorrhagic 19	Severe	3–12 months	GF, FM, WMST, RT	NA	Randomized, parallel groups	1 Hz rTMS over cM1/iTBS over iM1	20	NA	Pos
Brodie et al. (2014)	15	Cortico-subcortical 5, subcortical 9	NM	Mild to moderate	>6 months	STT, 2PD, WMFT, BB, GF	STT	Randomized, parallel groups	5 Hz rTMS over iS1	Five	1 day	Mix
Rose et al. (2014)	19	NM	Ischemic	Moderate	>6 months	WMFT, FM, ARAT, GF, PF, MAS, MAL	Functional Task Practice	Randomized, parallel groups	1 Hz rTMS over cM1	16	30 days	Neg
Motamed Vaziri et al. (2014)	12	NM	NM	Severe	>2 months	BI, FM	Standard physical therapy	Randomized, parallel groups	1 Hz rTMS over cM1	10	NA	Pos
Wang et al. (2014)	44	Cortical 16, subcortical 28	Ischemic 29, hemorrhagic 15	Moderate to severe	3–12 months	MRC, FM, WMFT	Standard physical and occupational therapy	Randomized, parallel groups	1 Hz rTMS over cM1, 1 Hz rTMS over cPMd	10	NA	Pos
**tDCS**												
Fregni et al. (2005)	6	Cortico-subcortical	NM	Mild to moderate	27.1 months	MRC, ASS, JTT	NA	Randomized, double-blinded, cross-over	ctDCS over cM1 and atDCS over iM1	One	NA	Pos
Hummel et al. (2005)	6	Subcortical 5, cortico-subcortical 1	Ischemic	Mild	44.4 months	MRC, FM, ASS, JTT	NA	Randomized, double-blinded, cross-over	atDCS over iM1	One	10 days	Pos
Hummel et al. (2006)	11	Subcortical	Ischemic	Mild to moderate	41.8 months	MRC, FM, ASS, sRT, PF	NA	Randomized, double-blinded, cross-over	atDCS over iM1	One	NA	Pos
Boggio et al. (2007)	9	Subcortical	NA	Mild to moderate	40.9 months	MRC, JTT, ASS	NA	Randomized, double-blinded, cross-over	ctDCS over cM1 and atDCS over iM1	5 days	2 weeks	Pos
Kim et al. (2010)	18	Cortical 5, subcortical 9, cortico-subcortical 4	Ischemic	Mixed	25.6 days	MRC (2–5) and FM (16–60), FM, BI	Occupational therapy	Randomized, parallel groups	ctDCS over cM1, atDCS over iM1	10	6 months	Mix
Lindenberg et al. (2010)	20	Cortico-subcortical	Ischemic	Severe	35.4 months	FM (20–56), WMF	Occupational therapy	Randomized, parallel groups	Bilateral tDCS	Five	1 week	Pos
Bolognini et al. (2011)	14	Cortical 9, cortico-subcortical 5	Ischemic 12, hemorrhagic 2	Moderate to severe	35.21 months	FM, BI, JTT, HG, MAL	CIT	Randomized, parallel groups	Bilateral tDCS	10	4 weeks	Pos
Hesse et al. (2011)	96	Mixed cortico-subcortical	Ischemic	Severe	0.93 months	BI, FM (<18), BB, MAS, MRC	Robot-assisted arm training	Randomized, parallel groups, multicenter	ctDCS over cM1 and atDCS over iM1	30 (6 weeks)	3 months	Neg
Madhavan and Stinear (2011)	9	Cortico-subcortical	NA	Lower extremity	130.8 months	FM-LE, dorsiflexion and plantar flexion movements	Tracking dorsiflexion and plantar flexion task	Randomized, cross-over	atDCS over iM1, atDCS over cM1	One	NA	Pos
Nair et al. (2011)	14	Cortical 9, subcortical 5	NA	Moderate to severe	31 months	FM (30.1)	Occupational therapy	Randomized, parallel groups	ctDCS over cM1	Five	1 week	Pos
Tanaka et al. (2011)	8	Subcortical	NA	Mixed, lower extremity	21.1 months	SIAS, knee extension, GF	Force knee extension	Randomized, cross-over	atDCS over iM1	One	NA	Pos
Stagg et al. (2012)	17	Cortical and subcortical	Ischemic 16, hemorrhagic 1	Mixed	37.9 months	FM, GF, response time task	NA	Randomized, double-blinded, cross-over	ctDCS over cM1 and atDCS over iM1	One	NA	Pos
Zimerman et al. (2012)	12	Subcortical	Ischemic	Mild	30 months	MRC, FM, ASS, FT	SFTT	Randomized, double-blinded, cross-over	ctDCS over cM1	One	3 months	Pos
Danzl et al. (2013)	8	NM	Ischemic 6, hemorrhagic 2	Moderate	1.1– 11.6 years	10 MWT, TUG, BBS, FAC, SIS	RGO-locomotor training	Randomized, double-blind, sham-controlled	atDCS over iM1	12	1 month	Pos
Giacobbe et al. (2013)	12	NM	NM	Moderate	>6 months	MRC	Robotic motor practice	Block-randomized, sham-controlled	atDCS over iM1	Four	NA	Mix
Khedr et al. (2013)	40	Cortical 18, cortico-subcortical 8, subcortical 14	Ischemic	Moderate	Anodal 13.8 ± 5.8, cathodal 12.3 ± 4.4, sham 12.6 ± 4.6 days	NIHSS, OMCASS, BI, MRC	Occupational therapy within 1 h after stimulation	Single-center, randomized, double-blind, sham-controlled	atDCS over iM1, ctDCS over cM1	6 days	1–3 months	Pos
Lefebvre et al. (2012)	18	Cortical 11, subcortical 7	Ischemic 16, hemorrhagic 2	Moderate	2.6 ± 1.5 years	PP, GF	Visuomotor learning task	Randomized, cross-over, sham-controlled, double-blind	Dual-tDCS	One	1 week	Pos
Rossi et al. (2013)	50	Cortical 3, cortico-subcortical 35, subcortical 12	Ischemic	Moderate to severe	2 days	FM, NIHSS	NA	Single-center, randomized, double-blind, sham-controlled	atDCS over iM1	Five	5 days, 3 months	Neg
Sohn et al. (2013)	11	Subcortical	Ischemic 4, hemorrhagic 7	Severe	63.00 ± 17.27 days	Static postural stability, isometric strength	NA	Randomized, cross-over, sham-controlled	atDCS over iM1	Two	NA	Mix
Au-Yeung et al. (2014)	10	NM	Ischemic 8, hemorrhagic 2	Mild to moderate	8.3 ± 3.2 years	PP, Stroop test	NA	Double-blind, sham-controlled, randomized, cross-over	atDCS over iM1, ctDCS over cM1	Three	NA	Mix
Fusco et al. (2014)	11	NM	Ischemic	Mixed	<30 days	CNS, BI, 9HPT, FM, TUG, 10MWT, 6MWT, RMI, FAC	Rehabilitative training	Double-blind, randomized, sham-controlled	ctDCS over cM1	10	30, 75–110 days	Neg
Lefebvre et al. (2014)	19	Cortical 8, subcortical 11	Ischemic 17, hemorrhagic 2	Moderate	4 ± 2 years	PP, PG	NA	Randomized, cross-over, sham-controlled, double-blind	Dual-tDCS	One	NA	Pos
O’Shea et al. (2014)	13	Cortical 6, subcortical 7	Ischemic 12, hemorrhagic 1	Moderate	1.5–5.8 years	sRT, FM, WMFT	NA	Cross-over, sham-controlled, single-blind	atDCS over iM1, ctDCS over cM1, Dual-tDCS	One	NA	Mix
Tahtis et al. (2014)	14	Cortical 6, subcortical 8	Ischemic	Moderate	2–8 weeks	TUG	NA	Double-blinded, sham-controlled, parallel design	Bi-cephalic tDCS	One	NA	Mix
Viana et al. (2014)	20	NM	Ischemic 19, hemorrhagic 1	Moderate	Anodal 31.9 ± 18.2, sham 35 ± 20.3 months	FM, WMFT, ASS, GF	Virtual reality therapy	Randomized, double-blind, sham-controlled	atDCS over iM1	15	5 weeks	Neg

*10MWT, 10 Meter Walk Test; 2PD, 2-point discrimination; 6MWT, Six-Minute Walking Test; 9HP, 9-holepeg Test; AI, Activity Index score; ARAT, Action Research Arm Test; ASS, Ashworth Spasticity Scale; BB, Box and Block Test; BBS, Berg Balance Scale; BI, Barthel Index; CIT, constrain-induced therapy; cM1, contralesional motor cortex; CNS, Canadian Neurological Scale; cPM, contralesional premotor cortex; cRT, choice reaction time task; FAC, functional ambulation category; FM, Fugl–Meyer Scale; FT, finger-tapping task; GF, grip force; iM1, ipsilesional motor cortex; JTT, Jebsen–Taylor Hand Function Test; MAL, motor activity log; MAS, Modified Ashworth Spasticity Scale; MI, motoricity index; Mix, mixed result; MRC, Medical Research Council; mRS, modified Ranking Scale; NA, not applicable; Neg, negative result; NIHSS, NIH Stroke Scale; NM, not mentioned; OMCASS, Orgogozo’s MCA scale; PA, pinch acceleration; PF, pinch force; PG, precision grip; Pos, positive result; PP, Purdue Pegboard Test; RMI, Rivermead Mobility Index; S1, primary sensory cortex; SFTT, sequential finger-tapping task; SIAS, Stroke Impairment Assessment Set; SIS, Stroke Impact Scale 16; sRT, simple reaction time task; SSS, Scandinavian Stroke Scale; STT, serial tracking task; TUG, Timed Up and Go Test; WMFT, Wolf Motor Function Test; WP, walking performance*.

## Potential Strategies and Underling Mechanisms to Increase the Effect of Non-Invasive Brain Stimulation

**Figure 2:**
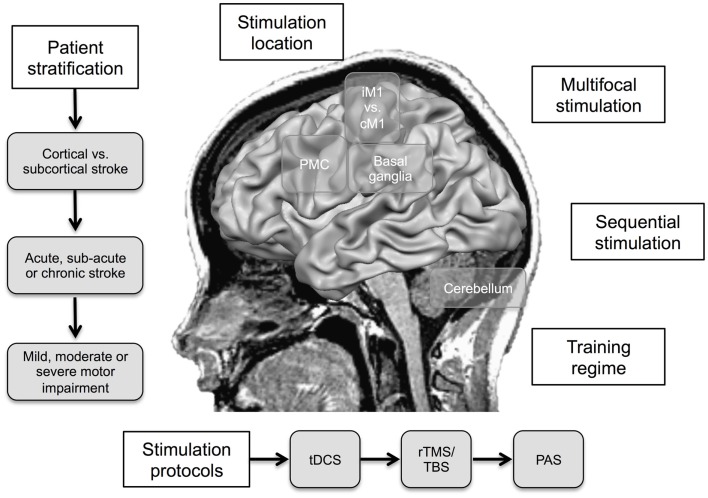
**Proposed strategies to increase the effect of non-invasive brain stimulation (NIBS) in motor recovery after stroke**. iM1, ipsilesional primary motor cortex; cM1, contralesional primary motor cortex; PMC, premotor cortex; tDCS, transcranial direct current stimulation; rTMS, repetitive transcranial magnetic stimulation; TBS, theta-burst stimulation; PAS, paired associative stimulation.

Effect sizes in the range between 8 and 30% of functional improvement in stroke patients have been reported for NIBS (Hummel et al., 2008b). In the next section, underlying principles and strategies to increase the effectivity are discussed (for illustration, see Figure 2; Table 2).

**Table 2:** **Pros and cons of potential strategies for increasing the effect of non-invasive brain stimulation**.

Strategy	Pros	Cons
Stimulation of iM1	• Direct enhancement of the reduced participation in the incompletely recovered motor network after stroke	• Higher risk of adverse effects due to induction of excitotoxicity in the penumbra and shunting of electrical current
Stimulation of cM1	• Stimulation of intact cortical areas	• Inhibitory stimulation might also impair complex motor function
Stimulation of secondary sensorimotor areas	• Stimulation of intact cortical areas	• More difficult to target
	• Modulation of cortico-cortical connections to M1	
Stimulation of the cerebellum	• Stimulation of intact cortical areas	• More difficult to target
	• Alternative target within the motor learning network	• Comparable high discomfort of cerebellar rTMS protocols
Simultaneous application of a motor training paradigm	• Simultaneous modulation of LTP-/LTD-like mechanisms	• Unfavorable homeostatic interactions
		• Not feasible for most rTMS protocols
Stimulation in the acute or sub-acute phase	• Enhanced adaptive plasticity	• Higher risk of adverse effects
Stimulation in the chronic phase	• More stable deficit	• Reestablished growth/plasticity inhibition
	• Lower risk of adverse effects	
Multi-session stimulation	• Enhancement of plasticity, e.g., induction of late-phase LTP/LTD-like neuroplasticity	• More complex and time-consuming
Multifocal stimulation	• Modulation of multiple nodes of the motor network	• Higher risk of adverse effects, e.g., shunting of current
	• Induction of additive or supra-additive effects	
Sequential stimulation	• Time-dependent modulation of multiple nodes of the motor network	• More complex setup
Patterned rTMS protocols	• Shorter delivery time	• Higher risk of adverse effects
	• Proposed potent modulatory aftereffect	• Need of a more complex and expensive setup
		• Mixed results

## Where to Stimulate?

Most studies conducted so far have focused on motor cortical stimulation. Both the iM1 and cM1 have been evaluated as targets. The advantage of stimulating iM1 is the possibility to directly enhance its reduced participation in the incompletely recovered motor network after stroke (Hummel et al., 2008b). Disadvantages are that the lesioned motor cortex might be more prone to adverse effects, for example, induction of excitotoxicity in the penumbra in the acute phase and the shunting of electrical current. Advantage of stimulating the cM1 is that NIBS is applied to intact cortical areas, especially relevant in patients with large almost complete lesions of iM1, thus the cM1 could here be used as a “window” to influence the motor system (Hummel et al., 2008b). A disadvantage is that inhibitory stimulation might also impair complex motor function of the paretic and the intact hand (Lotze et al., 2006). An alternative approach could be the stimulation of secondary sensorimotor areas, basal ganglia and cerebellum; for a conceptual review, please see Plow et al. (2014). First proof-of-principle studies provided promising results for SMA (Vollmann et al., 2013) and cerebellar (Galea et al., 2011; Wessel et al., 2015) stimulation combined with motor learning paradigms in healthy volunteers. In addition, recent studies demonstrated that inhibitory rTMS stimulation over cPMd (Wang et al., 2014) and the cerebellum (Kim et al., 2014b) improved motor function in stroke patients. Furthermore, excitatory rTMS over the ipsilesional primary sensory cortex (iS1) in chronic stroke facilitated learning of a Serial Tracking Task (Brodie et al., 2014).

## When to Stimulate?

In addition, the timing of the stimulation is most likely crucial for efficacy in motor rehabilitation. Since NIBS and motor learning share in part similar mechanisms, it has been proposed that simultaneous application could be more or perhaps only effective (Hummel et al., 2008b; Bolognini et al., 2009). Stagg et al. (2011) systematically addressed this issue focusing on tDCS. In their study, anodal tDCS applied during an explicit sequence-learning task was associated with faster learning, whereas cathodal tDCS and anodal tDCS applied prior to the learning task was associated with slower learning (Stagg et al., 2011). This concept might hold true for stroke patients. In a recent single-center randomized, double-blind, sham-controlled study anodal tDCS applied to iM1 without conjunctive motor training could not elicit significant differences in Fugl–Meyer motor scores in-between stimulation groups in 50 acute stroke patients (Rossi et al., 2013). In this regard, it can be speculated that only concomitant combination of motor training with NIBS leads to additive or even supra-additive longer-lasting effects.

A remaining crucial question is in which state, acute, sub-acute, or chronic phase of the recovery process NIBS should be applied. To date, this issue cannot be answered sufficiently, but some theoretical consideration can be drawn. A benefit of early stimulation could be the enhanced adaptive plasticity in the acute and sub-acute phase (Ward, 2004; Nudo, 2006; Hummel et al., 2008b). Whereas, an advantage of late stimulation could be the lower risk of interfering with rescue of critically nourished neurons and of inducing neuronal toxicity after glial scar formation in the chronic phase (Hummel et al., 2008b). Moreover, when chronic patients show a more stable deficit, it is easier to evaluate possible behavioral effects of NIBS protocols (Hummel et al., 2008b). To conclude, more systematic studies are needed to find the optimal time point for application of a plasticity-inducing protocols.

## How to Stimulate?

For methodological reasons, rTMS protocols, where subjects have to sit still during the intervention and cannot perform intensive motor training, are usually applied in an offline approach, utilizing the induced after-effects in the order of 30–60 min (Ziemann et al., 2008).

To increase effectivity, a promising complementary strategy could be the application of NIBS in a multi-session design. The magnitude of motor improvement in chronic stroke patients was increased over time by repetitive daily rTMS sessions (Fregni et al., 2006). In a complementary study, daily cathodal tDCS sessions to cM1 resulted in an augmented motor improvement when compared to a single-session design. Interestingly, this additive effect was not apparent in a design with weekly sessions (Boggio et al., 2007). An alternative compelling approach could be the use of spaced stimulation patterns, with multiple daily sessions. This might lead to prolonged after-effects via late-phase LTP/LTD-like neuroplasticity (Goldsworthy et al., 2014).

Lindenberg et al. (2010) have tested the effect of multifocal stimulation. In their study, they paired bihemispheric tDCS (anodal to iM1 and cathodal tDCS to cM1) with simultaneous occupational therapy (Lindenberg et al., 2010). Bihemispheric real stimulation resulted in a significant greater improvement in motor function when compared to sham stimulation. Although, an additive effect in motor function scores has been proposed, it remains unclear whether the bilateral approach is superior to a unilateral due to lack of sufficient control conditions. Sequential stimulation of different areas involved during the motor learning process could be an alternative strategy (Grimaldi et al., 2014).

Finally, a promising new interventional approach is the use of patterned rTMS protocols, like TBS. Proposed advantages are shorter delivery time and potent modulatory after-effects (Edwardson et al., 2013). In a first small proof-of-principle study, iTBS applied to iM1 improved motor function in chronic stroke patients (Talelli et al., 2007). In a larger semi-randomized clinical trial, in which iTBS to iM1 was combined with physical therapy over 10 days, this adjuvant effect of iTBS could not be replicated (Talelli et al., 2012).

Lastly, we want to point out that despite its similarities, rTMS, tDCS, and PAS differ in spatial and temporal resolution and the underlying neurophysiological mechanisms (Gandiga et al., 2006; Ziemann et al., 2008).

## Enhancement of Motor Learning by Innovative Training Regimes

In addition to adjuvant NIBS, stroke patients could potentially benefit from the use of innovative training regimes.

In a standard neurorehabilitative training session, usually a variety of different skills are practiced. The consolidation of the learned skills is highly dependent on the practice protocols. Performance during training is typically better, when the different skills are trained in a sequential order – block design. A random, intermingled training usually results in greater offline learning. This effect is called contextual interference (Battig, 1972). Recent fMRI studies provided evidence that random practice results in greater neuronal activity in regions crucial for preparation and production of learned motor skills at the end of training relative to block practice. It has been suggested that this persistent active preparation benefits offline learning (Wymbs and Grafton, 2009). Schweighofer et al. (2011) investigated the contextual interference effect in sub-acute stroke patients. In their study, patients with normal visuospatial working memory showed less long-term forgetting in a visuomotor task in the random training condition. Whereas, patients with low visuospatial working memory exhibited little long-term forgetting in both conditions (Schweighofer et al., 2011).

It has been suggested that the feedback given on a training session modulates its memory formation. This has been extensively investigated in animal models using associative learning paradigms. It was shown that reward of good performance and punishment of bad performance can modulate its memory consolidation (Tempel et al., 1983). Abe et al. (2011) recently studied this phenomenon in human motor skill learning in young healthy subjects. Learning under reward conditions enhanced long-term retention via significant offline memory gains. Whereas, training under neutral and punished conditions exhibited offline memory losses (Abe et al., 2011). Proposed neuroanatomical substrates of reward processing are dopaminergic neurotransmission (Zald et al., 2004) and in interconnected network involving the orbitofrontal cortex, amygdala, and ventral striatum (O’Doherty, 2004). Interestingly, a recent study provided evidence that high reward can facilitate learning of an ankle robotics motor training task in chronic stroke patients (Goodman et al., 2014).

## Future Directions

Non-invasive brain stimulation techniques are a promising adjuvant strategy for enhancing post-stroke recovery. However, many open questions remain. Future studies have to investigate possible mechanisms, the optimal site, time point and type of stimulation (Hummel et al., 2008b). Since stroke is a heterogeneous condition, it is very likely that patients will benefit most from individually tailored stimulation protocols. This could be achieved by patient stratification in regards to the clinical deficit, lesion location, lesion size, comorbidities, time in the recovery process, age, and gender (Hummel et al., 2008b). Also standardized algorithms, e.g., the PREP algorithm recently proposed by Stinear et al. (2012), could be useful for patient stratification. Another promising strategy could be the combination of different stimulation techniques. For instance, the combination of PNS and tDCS to iM1 in chronic stroke patients resulted in superior performance in a sequential motor task compared to unimodal stimulation or training alone (Celnik et al., 2009).

Overall, there is a great need for the development and testing of novel innovative interventional strategies individually tailored to the patients’ prerequisites. To achieve these goals, mechanism driven proof-of principle studies and large, multicenter, placebo-controlled trials are still necessary. Furthermore, a close cooperation between basic researchers and clinicians is needed to develop the field and bring innovative ideas from bench to bedside into daily clinical life.

## Limitations of Non-Invasive Brain Stimulation

In spite of the encouraging perspective of NIBS, limitations and unresolved issues remain.

A recent study by Wiethoff and colleagues emphasized the high in-between subject variability of tDCS protocols. They applied 10 min of tDCS over M1 in 53 healthy subjects in a cross-over design and assed the corticospinal excitability by measuring the MEP amplitude. At group level, anodal tDCS facilitated the MEP whereas cathodal tDCS showed no significant effect. In inspection of the single subject data, only 36% showed the expected facilitatory anodal and inhibitory cathodal effect, 21% showed a reversed pattern, 38% showed a polarity independent facilitatory, and 5% an inhibitory effect (Wiethoff et al., 2014). In this regard, several factors, which influence the response to plasticity-inducing NIBS protocols, like the history of synaptic activity, genetic polymorphisms of neurotrophins, use of CNS-active drugs, attention, age, gender, circadian rhythms, aerobic exercise, among others have been identified (Ridding and Ziemann, 2010).

It is of note that NIBS can potentially induce adverse effects, like headache (occasionally), skin burning (rare), seizures (very rare), and eyelid myokymia (very rare) (Brunoni et al., 2011; Wessel et al., 2013).

## Conflict of Interest Statement

The authors declare that the research was conducted in the absence of any commercial or financial relationships that could be construed as a potential conflict of interest.
